# Competition between Second-Generation Ethanol and Bioelectricity using the Residual Biomass of Sugarcane: Effects of Uncertainty on the Production Mix

**DOI:** 10.3390/molecules24020369

**Published:** 2019-01-21

**Authors:** Lucio Guido Tapia Carpio, Fábio Simone de Souza

**Affiliations:** 1 Energy Planning Program—PPE/COPPE, Federal University of Rio de Janeiro, Rio de Janeiro 21941972, Brazil; 2 Federal Center for Technological Education Celso Suckow da Fonseca—CEFET/RJ, Rio de Janeiro 2027110, Brazil; fabio.simone.souza@gmail.com

**Keywords:** biomass of sugarcane, second-generation ethanol, bioelectricity, sugarcane distillery, techno-economic analysis

## Abstract

Several economies around the world are using second-generation (2G) ethanol produced from agricultural residues, like sugarcane straw and bagasse, as a sustainable solution to replace petroleum products. Since first-generation (1G) ethanol uses the sugars of sugarcane, an integrated 1G–2G production would enable the production of more ethanol from the same amount of sugarcane without leading to increased use of arable land. The ethanol production process is complex, involving different high-energy consumption operations such as evaporation and distillation. The economic competitiveness of this process depends heavily on the amount of thermal and electrical energy produced using sugarcane straw and bagasse as input. Thus, the objective of this study was to use the mean-variance methodology to determine the optimal allocation of residual sugarcane biomass between 2G ethanol and bioelectricity productions, with simultaneous objectives of maximizing the return and minimizing the risk for investors of this sector. In this paper, four scenarios are analyzed. The first one is the base scenario that represents the current state of production costs and investments. scenarios 2, 3, and 4 considered four cuts of 10%, 20%, and 40% in the production cost of ethanol 2G, respectively. The results show the optimum biomass allocations and the growth rates of returns as a function of risk growth. It can be concluded that from scenario 4, the production of 2G ethanol becomes financially advantageous for the investor, presenting greater returns with smaller risks.

## 1. Introduction

The sugarcane industry is mainly known for producing ethanol and sugar. Sugar is consumed as a sweetener worldwide, while ethanol can be used as a renewable fuel, which is a promising substitute for non-renewable fossil fuels.

Debnath et al. [1] analyzed the possible complementary and substitutive effects between gasoline and ethanol demands to determine whether ethanol is a possible substitute for fossil fuel-derived products, considering the demands for ethanol are related to oil prices and the available legislation for biofuel production.

Currently, first-generation (1G) ethanol is the main liquid biofuel produced worldwide, with a global production of more than 97.80 billion liters per year. The main producers are the United States and Brazil, with a global participation of 58% and 27%, respectively [2]. Ethanol is mainly produced from corn in the Unites States. For this reason, there are concerns about the present and future competition between ethanol production and food consumption, which may lead to an increase in the prices of agricultural commodities, consequently causing famine not only in the USA, but also in corn-exporting countries. Brazilian ethanol is mainly produced from sugarcane, whose use is split between the production of sugar and 1G ethanol. As sugar is not a staple food like corn, the use of sugarcane is not perceived as a threat to the world food situation. However, there are concerns about the expansion of arable land use [3]. This threat can be mitigated by using the lignocellulosic biomass of sugarcane (bagasse, straw and tips) to produce second-generation (2G) ethanol, which would increase ethanol production without increasing land use. There is, however, a new dilemma because the lignocellulosic biomass of sugarcane can also be used in the production of bioelectricity through co-generation in the same power plant.

Traditionally, in addition to producing 1G ethanol and sugar, the sugarcane mills produce energy from the burning in boilers of the bagasse obtained after the sugarcane grinding, generating bioelectricity and steam [4].

With the technological advance, mills in the sector have the use of biomass of sugar cane (bagasse, straw and tips) as an alternative to produce lignocellulosic ethanol (2G ethanol), with the advantage of increasing the ethanol production by using the same cultivated area of sugarcane. In addition, there is the possibility of integrating the production of 1G ethanol, 2G ethanol and bioelectricity in the same plant [5,6,7]. The production of 2G ethanol starts with the pretreatment phase, followed by enzymatic or acidic hydrolysis, fermentation and finally distillation. Pretreatment is the most important phase where cellulose accessibility increases with increasing pre-treatment severity [8].

Several studies have shown the cost of each phase for producing 2G ethanol has an economically relevant impact and there is a prospect of a fall in the production costs of 2G ethanol [9,10,11,12]. Given that sugarcane bagasse can be used as input to produce 2G ethanol or bioelectricity, the sugarcane sector must decide what should be the most profitable destination of the bagasse available at the mill. To assist in this complex decision, there are already some proposals with different methodologies [13,14].

The objective of the present study is to present a new model with the objective of dimensioning the percentage of lignocellulosic material (LM) that should be assigned to each product in a portfolio formed by 2G ethanol production, production of electric energy to be commercialized in the regulated market, and production of electric energy to be commercialized in the free market. For this, we adapted the probabilistic Mean-Variance model proposed by Markowitz [15], which optimizes the risk-return ratio and considers the uncertainties of prices in the markets of products that comprise the portfolio. Complementarily, the net present value (NPV) of the considered activities was analyzed with the purpose of dimensioning the financial result of each of these activities and comparing the advantages of each scenario analyzed.

Initially, the Base scenario will be shown considering the current cost of production of 2G ethanol. The expectation of industry experts is that the cost of production of 2G ethanol be reduced by up to 40% in relation to the current costs. Therefore, three other possible scenarios for a gradual reduction of the production cost of 2G ethanol will be defined to measure the effects of allocating LM for each of the products in the portfolio. The initial reduction is 10% and 20%, followed by 40% (a situation in which 2G ethanol becomes financially advantageous in relation to the commercialization of electricity in both free and regulated markets).

## 2. Methods and Data

### 2.1. The Sugarcane Industry

**Figure 1:**
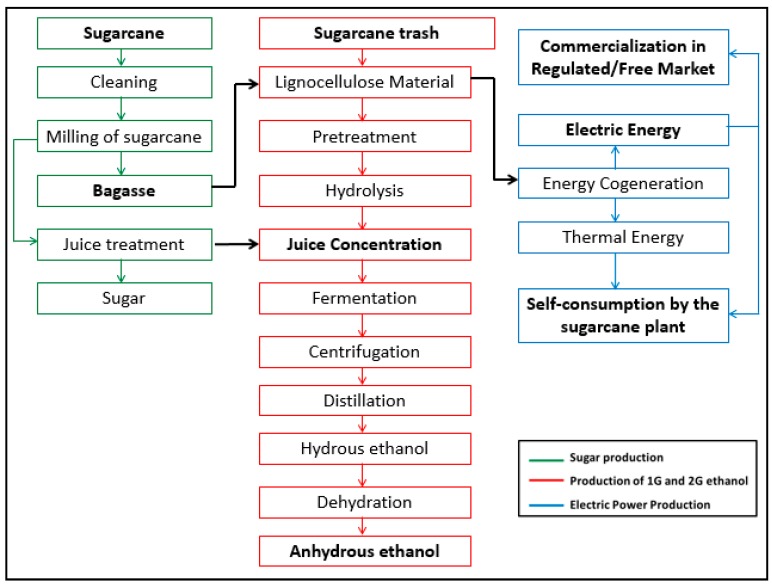
Flow chart of the production process in a sugarcane plant. Source: Own elaboration.

Figure 1 shows the flow chart of the production process in a sugarcane plant. The milling process produces sugarcane juice rich in fermentable sugars and bagasse as the residue. The bagasse obtained after milling and the straw collected from the field post-harvest is used as the LM, which is burned to produce electrical and thermal energy. Alternatively, the LM can be pre-treated to produce juice, which is added to the juice obtained from the milling process. This results in an aggregated amount of juice from which it is possible to produce a higher volume of ethanol without expanding the agricultural area of the sugarcane industry. The electricity produced from the burning of LM can be totally or partially commercialized in the free or regulated market, which generates extra revenue for the sugarcane plant owner. The electricity produced can also be used to run the activities in the plant.

For the process of production of 2G ethanol, as shown in Figure 1, after the arrival of the biomass in the plant the pre-treatment phase begins. According to Kim et al. [16], pre-treatment methods can be divided into two different categories: physical and chemical pretreatment methods and biological pretreatment methods. The purpose of pretreatment is to break down the structure of the lignocellulosic biomass to improve enzymatic digestibility and thus to enable the efficient bioconversion of monomeric sugars to biochemicals. Thereafter, the solid product obtained in the pre-treatment phase passes through enzymatic or acidic hydrolysis, then fermentation and finally distillation.

Sun and Cheng [17] emphasized that enzymatic hydrolysis is less expensive than acid hydrolysis and produces a higher yield. In addition to these characteristics, this form of catalysis is performed under mild temperatures (45 to 50 °C) and pH conditions. Enzymatic hydrolysis allows the union of the fermentation stage with the saccharification stage (SSF), thus lowering costs [18]. In the production of 2G ethanol, ML hydrolysis and fermentation steps can occur in the same reactor (SSF process).

This study is focused on the production of 2G ethanol through hydrolysis and fermentation of LM using the same reactor. This process is termed Simultaneous Saccharification and Fermentation (SSF). The ethanol produced is qualified as anhydrous or hydrous and then commercialized in their respective markets. Owing to the energy demands for various production activities, the thermal and mechanical energy produced by the cogeneration system in the sugarcane plant can be used to operate various systems in the plant such as control, lighting, and pumping systems.

Although the present work is based on the Brazilian sugarcane sector, the same approach is applicable to other agricultural crops around the world. In the next subsections will be presented the characteristics of the Brazilian sugar-alcohol industry and the description of an electric power cogeneration plant, showing the current technological and financial parameters for the production of 2G ethanol and bioelectricity.

#### 2.1.1. Cogeneration in a Brazilian Sugarcane Plant

In Brazil, there are various types of energy demand for the different stages of the production process in a sugarcane plant. At present, the average thermal energy consumption is 2.5 bar of steam per ton of processed sugarcane, whereas the average mechanical energy consumption is 16 kWh per ton of processed sugarcane, which is used in the preparation and milling of sugarcane. The average electric energy for own consumption is 12 kWh per ton of processed cane.

According to Dias et al. [7], the average straw produced is 140 kg per ton of harvested sugarcane, where half of the materials remain on the soil after harvest; these materials are used for soil fertilization, which facilitates soil recovery and increases soil productivity. The remaining materials are collected for use in sugarcane plants, which are either burned in the boilers or lumped together with sugarcane bagasse as LM to produce additional volumes of ethanol.

According to the Brazilian Sugarcane Industry Association (UNICA) [19], the percentage of sugarcane bagasse availability is estimated to be 25% per ton of ground sugarcane. The bagasse (sugarcane residues) produced from the milling process is added to the straw collected from the field post-harvest to be used as LM in the sugarcane plant. The use of bagasse for production activities in the sugarcane plant does not incur additional costs, even though the straw collected from the field incurs costs related to transportation, cleaning, and treatment, which amounts to US $9.38 per ton of straw [14]. The calorific values for sugarcane straw and bagasse used for electricity production are 12.96 and 7.57 MJ/kg, respectively [5].

#### 2.1.2. Technical Data Used in the Sugarcane Plant Optimization Model

In this work, we assumed that the volume of bagasse produced is 250 kg per ton of processed sugarcane and the percentage of straw produced (dry basis) is 14% per ton of sugarcane harvested [7]. The bioelectricity produced using the LM is 0.19 MWh per tonne of sugarcane [20], assuming that only 32% of LM is used for bioelectricity production. Then, the bioelectricity productivity is 0.59 MWh per ton of ML. The productivity of 2G ethanol is 158 L per ton of LM [6].

We assumed that the sugarcane plant operates using a condensing turbine with controlled steam extraction, and the condensing turbine works only during the harvest period. The sugarcane plant uses a boiler with a pressure of 100 bar for its production activities, where the boiler reaches a temperature of 530 °C. The extraction-condensing turbine is designed for bioelectricity generation. The bioelectricity produced is a function of the enthalpy drop and efficiency, which it is calculated based on the fundamental concepts of thermodynamics [21]. Assuming that the turbine only operates during the harvest period, the potential power of the turbine is 122 MW [14]. The surplus of electricity for exportation is determined from the difference between the total energy produced and total energy consumption of the sugarcane plant.

The technical data used in the optimization model are shown in Table 1.

#### 2.1.3. Financial Data Related to the Activities in the Sugarcane Plant

In an investment appraisal, the feasibility of a project is related to its capacity to generate profitable cash flow throughout its lifetime for a given investment and interest and discount rates for taxes associated with the project risk. The equivalent annual annuity (EAA) and equivalent annual cost (EAC) are the methods are used to evaluate the feasibility of the project with respect to the NPV, as the projects can be analyzed by the discounted payback, beneficiary cost index, and IRR. In the EAC method, the cost of an investment project is based on the project lifetime and discount taxes, provided that the project outcomes are related to the discount taxes.

To determine the EAC, the fixed costs and variable costs are deflated by the extended national consumer price index (known as the IPCA in Brazil), which were updated as of November 2018. Thus, the costs and historical data of the product prices are within the same temporal base. The costs and product prices for 2018 were used in this study, as shown in Table 2. 

In addition to the aforementioned costs, electricity tariffs were included in this work. According to the Brazilian Electricity Regulatory Agency (ANEEL, Brasília, Brazil) [22], the follow fees apply to this service: Tariff for Usage of electricity Transmission System (TUST, acronym in Brazil) at US $2.53/kW; and the Tariff for Usage of the electricity Distribution System (TUSD, acronym in Brazil) at US $1.24/kW.

Based on the definition of discount tax used in ventures of the sugarcane industry, the interest rate imposed by the Brazilian Development Bank (BNDES) is chosen as reference. The BNDES FINEM financing line will be used for the acquisition of 80% of the capital assets for the venture. The cost of equity to acquire the equipment for electricity production was 12% per annum [14] while the cost of equity to acquire the equipment for 2G ethanol production was 16% per annum. Consequently, the weighted average cost of capital (WACC) included 80% third-party capital and 20% equity capital, the WACC used for electricity generation was 9.34% per annum, and the WACC used for 2G ethanol production was 10.54% per annum.

#### 2.1.4. Equivalent Annual Costs (EAC) of 2G Ethanol and the Bioelectricity

As the Equivalent Annual Costs (EAC) enable the appraiser to determine the annual cost of owning, maintaining, and operating an asset annually throughout the project lifetime, the EAC is typically used as a decision-making indicator for investment projects, especially when there are uncertainties in the revenue to be generated from the investment. This is because the EAC provides an equalized assessment throughout the project lifetime, which serves as the basis to compare the financial capacity between multiple investment alternatives. The EAC, is an “equal apportionment per unit of time of the investment, opportunity costs, and operational costs of the alternatives” [23]. Table 3 and Table 4 show the EAC values for the base scenario of the 2G ethanol and bioelectricity.

As the experts in the sugarcane industry have estimated that the production cost of 2G ethanol will decrease in the future by up to 50% relative to the base scenario, the EACs per unit of 2G ethanol produced were determined for four probable future scenarios and the results are shown in Table 5. 

### 2.2. Market Prices of the Products in the Sugarcane Industry

In Brazil, the market prices of electricity sold in the regulated market are obtained from the electric energy auctions operated by the Chamber for the Commercialization of Electric Energy (CCEE) [24]; on the other hand, the electricity sold in the free market is obtained from spot prices [25].

The market prices for ethanol used in this work are obtained from the historical data of anhydrous ethanol compiled by the Center for Advanced Studies on Applied Economics (CEPEA-ESALQ/USP) [26] at the University of São Paulo, Brazil. It shall be noted that only the market prices from July 2003 to November 2018 are considered in this work.

**Figure 2:**
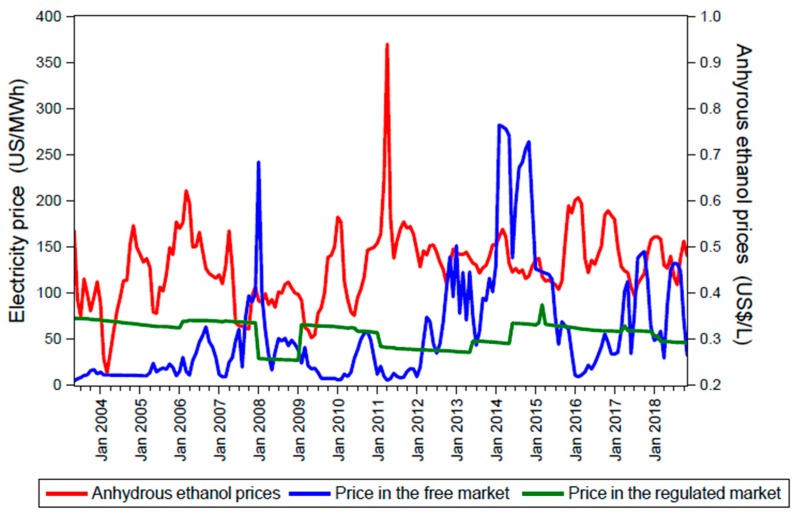
Historical data of the market prices for anhydrous ethanol and electricity in Brazil. Source: Own elaboration based on the data obtained from [24] and [26].

Figure 2 shows the market prices for electricity and anhydrous ethanol from July 2003 to November 2018. The market prices for electricity in both free and regulated markets are presented in US$/MWh whereas the market prices for anhydrous ethanol are presented in US$/L.

The market prices for anhydrous ethanol are primarily influenced by the volume of sugarcane produced during the harvest period, allocation of sugarcane during the production process in the sugarcane plants, sales of flexible-fuel vehicles, and dynamics of the market (buying and selling). 

In Brazil, currently, there are two environments for electricity contracts: (1) free contracting environment (abbreviated as ACL in Portuguese) and (2) regulated contracting environment (abbreviated as ACR in Portuguese). In the free contracting environment, the market price for electricity is negotiated directly between the interested parties, where each party assumes all of the risks associated with the contract. In the regulated contracting environment, commercialization of electricity is organized by the CCEE through auctions.

### 2.3. Base Scenario

To determine the monthly returns of each of the assets of the portfolio being analyzed, the equations for the definition of the Expected Return for ethanol and for the electric energy are used. Noting that LM is used as the inputs for electricity generation, the expected return can be determined based on the market price for electricity in the free or regulated market, the EAC per unit of electricity generation (US$ 37.81/MWh), and the amount of electricity generated per ton of LM, as shown in Equation (1).

(1) $$RETURN_{ENERGY}=(PRICE_{ENERGY}-37.81)\times 0.59$$

Likewise, the expected return for 2G ethanol production can be determined based on its market price, the EAC per unit of 2G ethanol produced (US$ 0.53/L), and the volume of 2G ethanol produced per ton of LM (158 L/tLM) used in the production process, as shown in Equation (2).

(2) $$RETURN_{ETHANOL}=(PRICE_{ETHANOL}-0.53)\times 158$$

### 2.4. Future Scenarios

Based on the base scenario defined previously, it is possible to expand the technological capabilities of 2G ethanol production due to the reduction in the production cost. In scenarios 2, 3 and 4, the production costs of 2G ethanol are reduced by 10%, 20% and 40%, respectively, considering the perspectives of industrial experts from UNICA [19]. The expected returns for 2G ethanol production for scenarios 2, 3 and 4 can be determined using Equations (3)–(5), respectively.

(3) $$RETURN_{ETHANOL.10\%}=(PRICE_{ETHANOL}-0.48)\times 158\;$$

(4) $$RETURN_{ETHANOL.20\%}=(PRICE_{ETHANOL}-0.43)\times 158$$

(5) $$RETURN_{ETHANOL.40\%}=(PRICE_{ETHANOL}-0.32)\times 158$$

### 2.5. Methodology for Optimal Allocation of Lignocellulosic Biomass

In order to determine the optimal portfolio of 2G ethanol and bioelectricity from the lignocellulosic biomass of sugarcane, it is important to consider the volatility of the returns of the assets of this portfolio, which bear a financial risk. Then, the investor in the sugarcane sector can make their decision according to their risk propensity.

Although there are currently several models available to optimize investments at different levels of sophistication, we present an adaptation of the mean-variance optimization model proposed by Markowitz [15], which is considered a benchmark for the financial market, to solve the problem of the sugar-energy sector proposed in this study. The justification for choosing this tool is due to the high relevance of the uncertainties of product prices in the decision-making process for the allocation of LM in the portfolio of these products. It is worth emphasizing that risk and return grow proportionately: the higher the expected return of the portfolio, the greater the risk of losses. Therefore, we considered that the investor of the sugar-energy sector is a rational agent who follows the following premises:

The investor evaluates its investments based on expected return and risk;

Between two assets with the same risk, the investor will always choose the one with the highest return; 

Whenever the investor has to choose between two assets with a same return, the investor will choose the one with the lowest risk;

The total assets are divisible, and the investor may allocate percentages for each product;

The set of portfolios preferable to all other possible portfolios forms an “efficient frontier”.

Markowitz uses the following parameters in order to characterize the model: (a) $R_{i}$, which denotes the return of asset i, (b) $x_{i}$ denotes the percentage of participation of asset i in the product portfolio, and (c) $\sigma_{ij}$ denotes the covariance between assets i and j. Using a bi-objective model to minimize portfolio risk (variance) and maximize expected return, we obtain the following model:

(6) $$\begin{array}{cc} Min\;\sigma_{C}^{2} & =\sum_{i=1}^{3}\sum_{j=1}^{3}x_{i}x_{j}\sigma_{ij}\\ Max\;E(R) & =\sum_{1}^{3}x_{i}R_{i}\\ subjet\;to & \\ & \sum_{i=1}^{3}x_{i}=1\\ & x_{i}\geq 0\;;\;i=1,2,3 \end{array}$$

Using the ε-restricted method [27], only one objective function is maintained; the other objective function is transformed into a new restriction, so the resulting model is:

(7) $$\begin{array}{ll} Min\;\sigma_{C}^{2} & =\sum_{i=1}^{3}\sum_{j=1}^{3}x_{i}x_{j}\sigma_{ij}\\ Subjet\;to: & \\ & \sum_{1}^{3}x_{i}R_{i}\geq \varepsilon \\ & \sum_{i=1}^{3}x_{i}=1\\ & x_{i}\geq 0\;;\;i=1,2,3 \end{array}$$

The value of the sugarcane plant is typically assessed based on the financial capacity of the production process. The financial capacity of the sugarcane plant can be assessed based on the NPV, which is obtained by the sum of cash flows (CF) in each period t that is discounted at a rate adjusted to the level of risk capital (k) and subtracted from the investment value (I) over the lifetime of the investment. When the Net Present Value (NPV) is positive, then the financial capacity is advantageous to carry out the investment [28]. This is one of the criteria that, together with the return and the risk of the efficient frontier, serve as the basis for the decision making for investments. The NPV [28] can be determined from Equation (8):

(8) $$NPV=-I+\sum_{t=1}^{T}\frac{CF_{t}}{{\left(1-k\right)}^{t}}$$

## 3. Results and Discussion

The optimum allocations of LM for electricity generation and 2G ethanol production in a representative sugarcane plant are determined using the Equations (6) and (7) presented in Section 2.5, based on the criteria that the sugarcane plant owner must commercialize the electricity generated in the plant in the free or regulated market and produce 2G ethanol.

**Figure 3:**
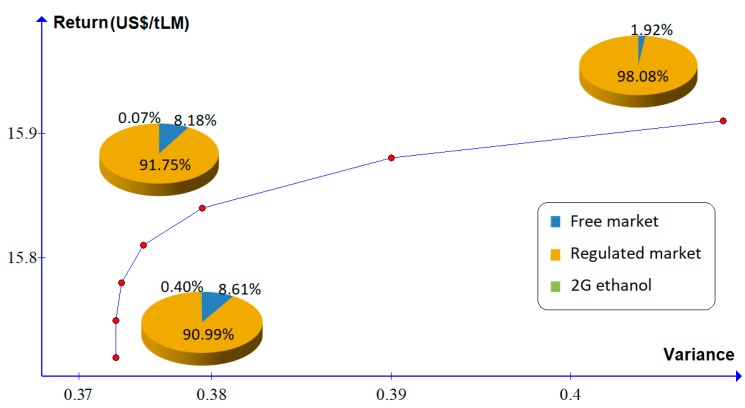
Efficient frontier for the base scenario.

For scenario 1 (base scenario), the minimum variance for the product portfolio is 0.3746 and the corresponding return is US$ 15.75 per ton of LM, as shown in Table 6. The expected return of the product portfolio increases with the increased risk (variance) of asset returns, as shown in Table 6 and Figure 3.

In this base scenario, the percentages of LM that should be allocated for generation of electricity to be traded in the regulated and free markets are 90.99% and 8.61%, respectively, and that only 0.40% of the LM should be allocated to the production of 2G ethanol.

Figure 3 shows that as the return increases, the percentage of LM allocation to the regulated market increases, albeit slightly, with the increased risk. Therefore, in this scenario, the best decision is to allocate LM to the production of electricity for the regulated market.

**Figure 4:**
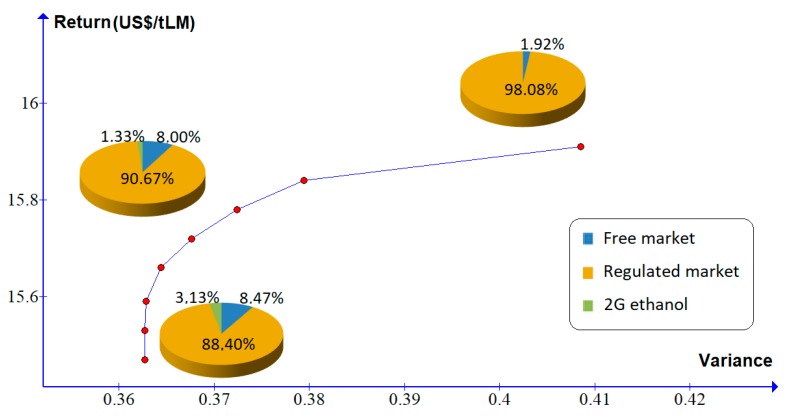
Efficient frontier for scenario 2.

In scenario 2, the production cost of 2G ethanol is reduced by 10% relative to the base scenario. In scenario 2, the production cost of 2G ethanol is reduced by 10% relative to the base scenario. In this case, a higher percentage should be allocated for electricity generation to be traded in the regulated and free markets (88.40% and 8.47%, respectively) and 3.13% of the LM should be allocated to the production of 2G ethanol. Figure 4 and Table 7 demonstrate that the percentage of LM allocated to the production of 2G ethanol decreases until it becomes insignificant. Therefore, in this scenario, only an investor very adverse to risk would invest in the production of 2G ethanol. 

**Figure 5:**
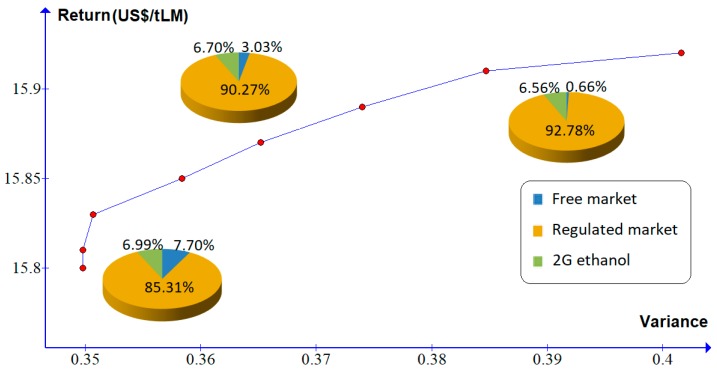
Efficient frontier for scenario 3.

In scenario 3, the production cost of 2G ethanol is reduced by 20% relative to the base scenario. In this case, a higher percentage should be allocated for electricity generation to be traded in the regulated and free markets (85.31% and 7.7%, respectively) and 6.99% of the LM should be allocated to the production of 2G ethanol (higher than in scenario 2). Figure 5 and Table 8 show that the percentage of LM allocated to the production of 2G ethanol slowly decreases with increased risk. Therefore, in this scenario, a risk neutral investor would make moderate investments in the production of 2G ethanol. 

**Figure 6:**
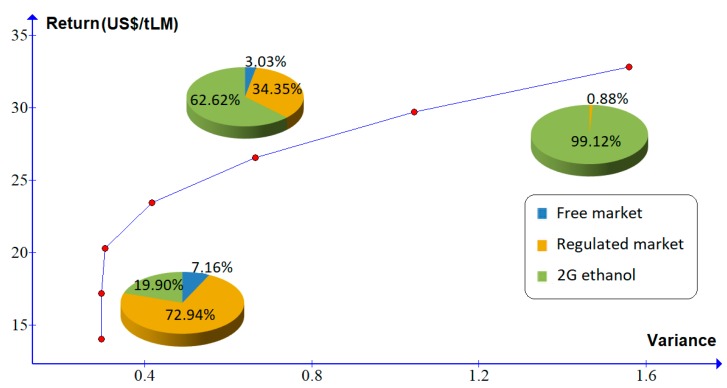
Efficient frontier for scenario 4.

In scenario 4, the production cost of 2G ethanol is reduced by 40% relative to the base scenario. In this case, the percentages that must be allocated for generation of electricity to be traded in the regulated and free markets are 72.94% and 7.16%, respectively, and the LM that must be allocated for the production of ethanol 2G is 19.90%, which is considerably higher than the percentage allocated in the previous scenarios. As can be seen in Figure 6 and in Table 9, the percentage of LM allocated for the production of 2G ethanol increases considerably for increases in risk. Therefore, in this scenario, it is advisable to invest in the production of 2G ethanol, the more prone to the risk is the investor, the higher should be the investment in the production of 2G ethanol.

Notably, as the cost of production of 2G ethanol decreased, there was a corresponding increase in the NPV, which started with a negative value. In scenario 4, the NPV reached a value of US $35,601,674.56 based on the average market price of anhydrous ethanol in November 2018. For this last scenario, the NPV is higher compared with that obtained by the sale of electricity in the regulated market or in the free market.

As a suggestion for future research, we propose to study a dynamic model, similar to the static Mean-Variance model proposed in the present work, which would allow us to generalize the results of the problem studied here, using a dynamic-stochastic programming model that analyzes the properties of convergence and stability for the economic growth of the sugarcane energy industry [29].

## 4. Conclusions

This work shows a new model to respond to the new challenge faced by investors in the sugarcane industry, i.e., the decision on the percentage allocation of LM of sugarcane between three alternatives: 2G ethanol, bioelectricity to be marketed in the regulated market or bioelectricity to be traded on the free market. The model presented considers the risks and uncertainties inherent in the sugar and alcohol industry, which are caused mainly by the volatility of prices in the different markets.

Four possible scenarios were studied, the first one being the base scenario that reflects the cost of producing 2G ethanol for 2018. For the other three scenarios, reductions of 10%, 20% and 40%, respectively, were considered in the production cost of 2G ethanol. This sequence of cuts is in line with the downward trend in cost of production, which has been the subject of different studies.

After analyzing the results of Section 3, we can conclude that, in the first three scenarios, the LM should be allocated mainly to the production of bioelectricity and should be commercialized in the regulated market. In these three scenarios the risk-prone investor, in order to increase his/her financial return, can diversify his/her stake by allocating a small percentage of LM for 2G ethanol production. This diversification is more plausible according to the reduction in the production cost of 2G ethanol.

When we analyzed the fourth scenario, where the production cost of 2G ethanol was reduced by 40% (cost of US$ 0.32 per liter), we conclude that LM allocation for 2G ethanol production becomes significant. In this scenario, the risk neutral investor should divide his/her share between 2G ethanol and bioelectricity to be commercialized on the regulated market, whereas the most risk-prone investor should prioritize the production of 2G ethanol to obtain a higher return.

Finally, a complementary analysis was carried out, observing the NPV values for the results of each scenario, concluding that in all of the scenarios, the NPV values for bioelectricity were positive. On the other hand, 2G ethanol started with a negative NPV in the first scenario, whereas the value increased in scenarios 2 and 3 until reaching the fourth scenario, with a financially advantageous NPV in relation to bioelectricity.

## Figures and Tables

**Table 1:** Technical data used in this study.

Sugarcane Processed	3 Mt Cane/Year
Harvest period	240 days
Percentage of time use	85%
Actual time of production	4896 h
Bagasse/sugarcane ratio (50% humidity)	25%
Sugarcane trash/sugarcane ratio (15% humidity)	14%
Total installed power	122 MW
Bioelectricity Productivity	0.59 MWh/tLM
Vapor pressure of entry	100 bar
Outlet steam temperature	530 °C
Ethanol 2G Productivity	158 L/tLM

sent 3 million tons of sugarcane processed per year, 0.59 megawatt-hour of bioelectricity produced per ton of LM, and 158 liters of 2G ethanol produced per ton of LM, respectively.

**Table 2:** Data used for financial analysis.

Data	Electric Power Generation (Rankine Cycle)	2G Ethanol Production
Useful life of equipment	25 years	20 years
Residual value of equipment	10% of initial investment	10% of initial investment
Value of investment	US$ 99,706,166.63	US$ 331,790,062.50
Operation and maintenance (O & M)	US$ 5,970,719.69/year	US$ 38,283,468.75/year
Operating Insurance	US$ 299,261.56/year	US$ 1,990,740.31/year
Cost for connection to the transmission network	US$ 5,043,750.00	-

Source: Own elaboration based on Dantas et al. [14] and Dias et al. [7].

**Table 3:** EAC values for 2G ethanol production in the base scenario.

Total EAC for 2G ethanol production	US$ 79,803,022.84
Total volume of 2G ethanol produced	151,680,000 L
EAC per unit of production	US$ 0.53/L

Source: Own elaboration.

**Table 4:** EAC values for bioelectricity production in the base scenario.

Total EAC for electricity generation	US$ 21,413,548.53
Total amount of electricity generated	566.400 MWh
EAC per unit of production	US$ 37.81/MWh

Source: Own elaboration.

**Table 5:** EACs per unit of 2G ethanol produced for different scenarios of production costs.

Scenario	Event	EAC per Unit of Production
Base scenario	No reduction of production cost (based on values in year 2018).	US$ 0.53/L
scenario 2	Production cost is reduced by 10%.	US$ 0.48/L
scenario 3	Production cost is reduced by 20%.	US$ 0.43/L
scenario 4	Production cost is reduced by 40%.	US$ 0.32/L

Source: Own elaboration.

**Table 6:** Results of the mean-variance model for the base scenario.

Return (US$)	Variance	Free Market	Regulated Market	2G Ethanol
15.72	0.3746	8.61%	90.99%	0.40%
15.75	0.3746	8.61%	90.99%	0.40%
15.78	0.3750	8.41%	91.34%	0.25%
15.81	0.3762	8.18%	91.75%	0.07%
15.84	0.3795	6.71%	93.29%	0.00%
15.88	0.3900	4.31%	95.69%	0.00%
15.91	0.4085	1.92%	98.08%	0.00%

Source: Own elaboration.

**Table 7:** Results of the mean-variance model for scenario 2.

Return	Variance	Free Market	Regulated Market	2G Ethanol
15.47	0.3627	8.47%	88.40%	3.13%
15.53	0.3627	8.47%	88.40%	3.13%
15.59	0.3629	8.38%	88.82%	2.80%
15.66	0.3644	8.19%	89.75%	2.06%
15.72	0.3676	8.00%	90.67%	1.33%
15.78	0.3724	7.81%	91.60%	0.59%
15.84	0.3795	6.71%	93.29%	0.00%
15.91	0.4085	1.92%	98.08%	0.00%

Source: Own elaboration.

**Table 8:** Results of the mean-variance model for scenario 3.

Return	Variance	Free Market	Regulated Market	2G Ethanol
15.80	0.3498	7.70%	85.31%	6.99%
15.81	0.3498	7.70%	85.31%	6.99%
15.83	0.3507	6.58%	86.50%	6.92%
15.84	0.3536	5.40%	87.75%	6.85%
15.86	0.3584	4.21%	89.02%	6.77%
15.88	0.3652	3.03%	90.27%	6.70%
15.89	0.3740	1.85%	91.52%	6.63%
15.91	0.3847	0.66%	92.78%	6.56%
15.92	0.4016	0.00%	96.23%	3.77%

Source: Own elaboration.

**Table 9:** Results of the mean-variance model for scenario 4.

Return	Variance	Free Market	Regulated Market	2G Ethanol
14.06	0.2969	7.16%	72.94%	19.90%
17.19	0.2969	7.16%	72.94%	19.90%
20.31	0.3049	6.59%	67.18%	26.23%
23.44	0.4181	4.82%	50.74%	44.44%
26.56	0.6650	3.03%	34.35%	62.62%
29.69	1.0457	1.27%	17.87%	80.87%
32.81	1.5603	0.00%	0.88%	99.12%

Source: Own elaboration.

## Abbreviations

The following abbreviations are used in this manuscript:

1G ethanol: First-Generation ethanol
2G ethanol: Second-Generation ethanol
ACL: Free Contracting Environment (in Portuguese)
ACR: Regulated Contracting Environment (in Portuguese)
ANEEL: Brazilian Electricity Regulatory Agency
BNDES: Brazilian Development Bank
BNDES FINEM: Banking product with financing lines
CCEE: Chamber for the Commercialization of Electric Energy (in Portuguese)
CEPEA/USP: Center for Advanced Studies on Applied Economics/University of São Paulo (in Portuguese)
EAA: Equivalent Annual Annuity
EAC: Equivalent Annual Costs
IPCA: Broad Consumer Price Index (in Portuguese)
IRR: Internal Rate of Return
LM: Lignocellulosic Material
NPV: Net Present Value
PLD: Price for Settlement of Differences (in Portuguese)
TUSD: Distribution Use of System Charge
TUST: Transmission Use of System Charge
SSF: Simultaneous Saccharification and Fermentation
UNICA: Union of the Sugarcane Industry (in Portuguese)
WACC: Weighted Average Cost of Capital
